# Research Progress in the Treatment of Complications and Sequelae of COVID-19

**DOI:** 10.3389/fmed.2021.757605

**Published:** 2021-12-02

**Authors:** Jinpeng Wang, Kuoyun Zhu, Yuchuan Xue, Guangfu Wen, Lin Tao

**Affiliations:** ^1^Department of Orthopedics, First Hospital of China Medical University, Shenyang, China; ^2^The First Department of Clinical Medicine, China Medical University, Shenyang, China; ^3^Department of Pediatrics, Shengjing Hospital of China Medical University, Shenyang, China

**Keywords:** COVID-19, complication, sequelae, eight major systems, treatment

## Abstract

With the improvement in the understanding of COVID-19 and the widespread vaccination of COVID-19 vaccines in various countries, the epidemic will be brought under control soon. However, multiple viruses could result in the post-viral syndrome, which is also common among patients with COVID-19. Therefore, the long-term consequences and the corresponding treatment of COVID-19 should be the focus in the post-epidemic era. In this review, we summarize the therapeutic strategies for the complications and sequelae of eight major systems caused by COVID-19, including respiratory system, cardiovascular system, neurological system, digestive system, urinary system, endocrine system, reproductive system and skeletal complication. In addition, we also sorted out the side effects reported in the vaccine trials. The purpose of this article is to remind people of possible complications and sequelae of COVID-19 and provide robust guidance on the treatment. It is extremely important to conduct long-term observational prognosis research on a larger scale, so as to have a comprehensive understanding of the impact of the SARS-CoV-2 on the human body and reduce complications to the greatest extent.

## Introduction

Since it was first reported in December 2019, the severe acute respiratory syndrome coronavirus 2 (SARS-CoV-2) is still prevalent globally (1). The number of people recovering from COVID-19 continues to rise, and this encouraging result gives people hope. However, are the survivors of COVID-19 really healthy? Could the human body completely return to normal when the viruses are cleared? Recent studies have shown that many patients with mild to moderate COVID-19 may become “long-term victims,” and one-third of mild patients will still suffer from lasting sequelae after recovery. It was also observed that 76% of COVID-19 survivors were troubled with at least one symptom at 6 months after symptom onset, which was even more common among females (2).

It is not clear why COVID-19 has these lasting effects. Many viruses can result in the so-called “post-viral syndrome,” which is described as health problems that persist after the viruses are cleared from the body. These are the result of inflammation or other damage that occurs when the immune system resists infection. The respiratory system is the primary target of SARS-CoV-2 which is transmitted mainly through the respiratory tract. The main clinical manifestations are related to the symptoms of pneumonia, characterized by severe acute respiratory distress syndrome (3). Meanwhile, almost all of the organs are at high risk of being attacked by SARS-CoV-2 (4, 5). Various unusual symptoms are observed in patients with COVID-19 which ranges across the body, such as thrombosis, multiple organ failure, immune defects and complications in the immune system. According to clinical statistics, the mortality rate of COVID-19 patients with acute kidney injury was three times higher than those without kidney injury and the mortality rate of patients with cardiovascular system complications is also relatively higher (6, 7). In addition, it is also common for COVID-19 patients with complications of the nervous system, digestive system, endocrine system, reproductive system and motor system (8–12).

Therefore, the prevention and treatment of the complications and sequelae of COVID-19 should be the focus in the post-epidemic era. In this study, we systematically summarize the treatment of complications and sequelae associated with eight major systems in patients with COVID. Additionally, we also sorted out the complications and sequelae reported in the vaccine trials. We believe that our research will help medical workers around the world respond more effectively to the COVID-19 in the post-epidemic era.

## Respiratory Sequelae

The respiratory system is the primary target of the SARS-CoV-2 attack. The main clinical symptoms of COVID-19 are related to pneumonia, with the characteristics of severe acute respiratory distress syndrome. In addition, it is noted that many patients who have recovered from COVID-19 still suffered from coughing and shortness of breath, and severe cases would even develop extensive pulmonary fibrosis, which could result in severe dyspnea. Supportive treatment is the basis for the treatment of respiratory symptoms in patients with COVID-19. It is important to let patients rest in bed and to provide them with good nutrition. Meanwhile, a timely infusion is necessary to maintain the stability of the internal environment, avoid electrolyte disturbances and ensure an acid-base balance. Oxygen therapy is used for mild and ordinary cases to relieve difficult breathing, while a ventilator is required to ensure unobstructed breathing for critical patients (13, 14). In addition, it was noted by Han X et al. that survivors with severe COVID-19 are at the high risk for post-COVID fibrosis, but there are still no effective therapeutic strategies for pulmonary fibrosis induced by COVID-19 (15, 16). Several anti-fibrosis drugs have entered clinical trials, such as Nintedanib (NCT04619680; NCT04541680 and NCT04338802) and Pirfenidone (NCT04282902 and NCT04607928). Meanwhile, IN01 vaccine (NCT04537130), a hybrid of the recombinant epidermal growth factor and cholera toxin B-subunit domain G33D, has also enter phase Ib clinical trials (16). In extremely severe patients, even if the endotracheal tube is intubated with pure oxygen (100% oxygen concentration), the partial pressure of oxygen in the body is very low, indicating that there is already severe respiratory failure. Hence, extracorporeal Membrane Oxygenation (ECMO) can be applied to carry out oxygenation through an artificial membrane allowing the lung to recover (17). The use of ECMO was recommended by the World Health Organization (WHO) for COVID-19 patients with severe cardiorespiratory failure (18). According to an international cohort study including 1,035 patients with COVID-19 who received ECMO support, use of ECMO was recommended among patients with refractory COVID-19-related respiratory failure (19). BI 764198 is a potent and selective TRPC6 inhibitor that can reduce lung damage in hospitalized patients with new coronary pneumonia and reduce the risk or severity of acute respiratory complication. A phase II clinical trial of BI 764198 was recently launched. Antimicrobial therapy is also needed while maintaining respiratory function. Antiviral therapy mainly uses anti-influenza and anti-AIDS drugs, but the effect is limited and there is no specific drug (20). At the same time, clinicians use antibacterial drugs to prevent potential risks of co-infection, which should be limited to patients with coinfections and those people developing healthcare-associated infections. Critical patients with severe lung fibrosis may require a lung transplantation. Additionally, the serum levels of antimicrobials of patients receiving antimicrobial treatment should be monitored strictly (21). In short, the treatment of respiratory complication and sequelae is a complicated process. The principle is to kill the pathogenic microorganisms and to maintain normal breathing.

## Cardiovascular Complication

Although SARS-CoV-2 attacks the respiratory system, it also has negative effects on cardiovascular systems (22). As such, patients with COVID-19 usually exhibited some clinical manifestations caused by cardiovascular diseases, including myocardial injury, myocarditis, acute coronary syndrome (ACS), acute myocardial infarction (AMI), cardiac arrhythmia and heart failure (23). At present, traditional anti-hypertension drugs are administered routinely to patients with a history of cardiovascular diseases during treatment for COVID-19 and they show no signs of aggravating the condition (24, 25). However, there are some key points to be considered. Concerns have been raised about the safety of ACE inhibitors (ACEi) and angiotensin receptor blockers (ARB) during the COVID-19 pandemic, which could upregulate the expression of ACE2 in many tissues, including cardiomyocytes. Since SARS-CoV-2 could bind to ACE2 to access human cells, there is a potential increased risk of developing COVID-19 or a more severe illness in patients who are already undergoing background treatment with ACEi/ARB. It was observed in a large multicenter cohort study that the prevalent treatment with ACEi/ARB among patients with hypertension is not associated with increased risk of COVID-19 diagnosis, hospital admission or subsequent complications (26). It was also observed in a retrospective study involving 362 patients with hypertension that ACEI/ARB had no significant effect on the severity and outcomes of COVID-19 (27). However, it was also noted that temporary discontinuation of ACEi or ARB had no appreciable effect on the maximum severity of disease within 30 days which, on the contrary, might be associated with a faster and better recovery (28). Therefore, appropriate strategies for the use of ACEi/ARB among COVID-19 patients with hypertension should be evaluated on individual basis such as the progression of diseases, indications for ACEi/ARB and the clinical feasibility of replacement therapies (28). In addition, various antiretroviral medications have significant interactions with cardiac drugs, which should be considered and an appropriate dose change also needs to be changed (29). ECMO is also recommend for patients with cardiac failure secondary to COVID-19 (30). However, coagulation abnormalities is common among critically ill patients with COVID-19, including thrombotic microangiopathy and venous and arterial thromboembolic complications (31). Meanwhile, ECMO usually leads to hypercoagulation status of patients. Hence, it is crucial to evaluate the coagulation status of COVID-19 patients who received ECMO and also dosages of anticoagulation drugs (32).

## Neurological Complication

Neurological symptoms, including meningitis, encephalitis, myelitis, acute disseminated encephalomyelitis and stroke, are also common in patients with COVID-19 and these patients might suffer from severe neurological sequelae (33). It is recommended for patients with cerebrovascular-related symptoms to use antihypertensive drugs, angiotensin-converting enzyme inhibitors (ACEI) and angiotensin II receptor blockers (ARB) in order to increase the expression of ACE2 (34). However, patients with hypertension may need to switch to calcium channel blockers (CCB) and diuretics (35). In addition, the patients with intracranial infections should be treated in terms of the principles of diagnosis and treatment of intracranial infection, protecting against dehydration, controlling seizures and providing antipsychotic treatment (36). In addition, sufficient attention and symptomatic treatment should be given for complication secondary to nerve damage, such as abnormalities of the skeletal muscle, glands and internal organs.

## Digestive Complication

The digestive system also presents a high risk of being invaded by SARS-CoV-2 (37). A wide range of digestive symptoms are observed in patients with COVID, including gastrointestinal reactions such as loss of appetite, vomiting, diarrhea and indigestion, as well as liver dysfunction (9, 38). Despite the presence of all of the digestive disorders associated with the coronavirus, to date there are no guidelines for the treatment of gastrointestinal symptoms associated with COVID-19. The intestinal flora produces various vitamins, fatty acids, bile acids and immune factors through the fermentation and decomposition of food, and it participates in the regulation of immune function (39). Significant alterations were observed in COVID-19 patients with characteristics of increased opportunistic pathogens and decreased beneficial commensals during hospitalization (40). In addition, it was also noted by Y. Wu et al. that gut microbiota was associated with SARS-Cov-2 virus load, and the microbiome dysbiosis caused by COVID-19 persists still exists even after the viral clearance (41). Therefore, strategies to restore the composition of intestinal flora might reduce the severity of COVID-19 and related complications. Indeed, the prevention and treatment strategies for COVID-19 considering gastroenterology and intestinal microbiota have been of extensive interest. It was proposed in multiple studies that treatment with probiotics might alleviate the progression of COVID-19 (42–44). However, it was demonstrated by Mak JWY et al. that there is no direct evidence for the use of probiotics in COVID-19, and conventional probiotic treatment is not appropriate among patients with COVID-19 (45). Hence, further studies should be focused on the pathogenesis of SARS-CoV-2 and its effect on intestinal microbiota. Meanwhile, whether probiotic treatment could be used as complementary resource for the prevention and restoration of alterations of intestinal microbiota in the absence of direct evidence still warrants further discussion. In addition, ACE2 inhibitors have been found to affect the composition of the intestinal flora and relieve gastrointestinal symptoms in mice by activating mTOR (46). Enteral nutrition can also help restore intestinal digestion, absorption and physiological peristalsis, and maintain the normal function of the gastrointestinal microecology and mucosal immunity. For patients with severe gastrointestinal symptoms of COVID-19, a nutritional risk assessment could be performed (47). Enteral nutrition is the preferred pathway for promoting intestinal integrity and immune function in high-risk populations, including critically ill patients (48). For patients with severe liver damage from COVID-19, drugs that protect the liver, reduce inflammation and reduce jaundice should be used, such as polyene phosphatidyl choline, glycyrrhizic acid, bicyclol, and vitamin E (49). One or two drugs should be selected according to the degree of liver function damage, to avoid aggravating the liver burden and drug interactions.

## Urinary Complication

After lung infection, SARS-CoV-2 enters the systemic circulation and reaches the kidneys, where it is concentrated and damages resident cells (50). It is observed that kidney involvement in patients with COVID-19 is frequent, which ranges from mild proteinuria to acute kidney injury (AKI) (51, 52). In addition, the mortality of patients with AKI is apparently higher than those without kidney damages (6). Therefore, it is necessary to consider all available therapies apart from regular antiviral medications to support renal function. Through daily monitoring and analysis of the patients, clinicians can take potential early intervention measures, including CRRT, as soon as possible to protect renal function, especially for patients with monotonously elevated plasma creatinine (Cre) levels, which may be the key to decrease mortality. Continuous renal replacement therapy (CRRT), a process that mimics the process of glomerular filtration, injects arteriovenous blood into a semipermeable member filter to non-selectively remove the overexpressed inflammatory cytokines from the circulation and block the inflammatory cascade reactions, which subsequently prevents the progression of the disease and improves the prognosis of patients with COVID-19. In addition, systemic anticoagulation therapy is also essential for patients receiving CRRT due to the hypercoagulable state of the patients and the high frequency of clotting in CRRT filters (52). The use of convalescent plasma of patients recovered from COVID-19 was traditionally considered to be capable of accelerating clinical recovery and thereby reduce the occurrence of kidney injury. However, it was noted in a multicenter, randomized clinical trial that compared with patients with standard treatment, no significant benefit was observed in clinical improvement at 28 days or mortality among patients who received standard treatment combined with convalescent plasma transfusion therapy (53). It was also observed in a randomized trial that convalescent plasma therapy in addition to standard treatment was not associated with decreased mortality rate or improvement of other clinical outcomes among patients with severe COVID-19. Recently, it was also implicated in a meta-analysis published in *JAMA* that convalescent plasma administration was not significantly associated with clinical improvement in all-cause mortality or other clinical outcomes (54). Hence, more clinical trials are acquired to establish the optimal conditions for the use of convalescent plasma. Additionally, kidney transplantation is the final choice if the patient's condition requires and permits it.

## Endocrine Complication

It is indicated that endocrine organs such as pancreas, thyroid, adrenal glands and pituitary are also vulnerable to SARS-CoV-2 (55). In addition, COVID-19 could aggravate insulin resistance. Patients with diabetes mellitus are more susceptible to SARS-CoV-2 and are associated with increased mortality (56). For patients with severe comorbidities, terminal illness, or inability to conduct frequent glucose monitoring or close nursing supervision, less aggressive insulin therapy aimed at reducing glucosuria, dehydration, and electrolyte disturbances may be more appropriate (57). Thyrotoxicosis may worsen cardiovascular disease and, in some cases, lead to arrhythmias. Therefore, rapid evaluation of free thyroid hormones and TSH contributes to early diagnosis and appropriate treatment and helps to avoid more serious complication. Moreover, The British Thyroid disorder Association and The Society for Endocrinology have issued a consensus statement on the specific problem of thyroid dysfunction during the COVID-19 pandemic, stating that patients with underlying hypothyroidism or hyperthyroidism are advised to continue taking their prescription drugs as usual (58). In addition, rapid screening of the function of the pituitary-adrenal axis and identification of this condition may lead to appropriate alternative therapy to prevent severe shock. In the presence of subacute thyroiditis or adrenal insufficiency, corticosteroid therapy should be used to interrupt the release of large amounts of thyroid hormone and improve adrenal function, thereby preventing the clinical deterioration of these patients (59).

## Reproductive Complication

There are also obvious gender differences in reproductive complication and sequelae of COVID-19. Symptoms including oligospermia, orchitis, scrotal discomfort and erectile dysfunction are observed in male patients (60–62), while premature birth, fetal distress and premature rupture of fetal membranes are observed in pregnant patients and fetuses with COVID-19 (63, 64). In addition, male patients infected with SARS-CoV-2 also exhibited impaired spermatogenesis. It was observed in an observational study that 39.1% of male patients with COVID-19 have oligospermia and 60.9% of them have a high inflammatory state in their semen (61). Persistent fever, which is common among COVID-19 patients, would result in temperature changes in the testes and subsequently lead to damage and degeneration of germ cells (65). There is no specific treatment for reproductive dysfunction in patients with COVID-19, but it is important to lower the body temperature to prevent impairment of spermatogenesis due to the fever. Phosphodiesterase-5 inhibitors have been traditionally approved for the treatment of erectile dysfunction since 1998. Surprisingly, sildenafl, the first PDE-5 inhibitor applied in the clinical, has been suggested as a treatment for COVID patients by improving pulmonary function and lowering the risk of vascular injury and thrombotic complication in COVID-19 patients (66). Hence, it is significant to evaluate the effect of other medical interventions on COVID-19 that have a protective function of the reproductive system to reduce the risk of reproductive complication after recovery.

## Skeletal Complication

Due to the respiratory system damage caused by SARS-CoV-2, there is an increase in blood lactic acid levels and decrease in blood cell oxygen-carrying capacity. Hence, skeletal muscle hypoxia and ischemia may occur in patients with COVID-19, resulting in myalgia and joint pain (67). In this case, restoring the body's oxygen level will help to relieve the pain. Therefore, when the body's viral load decreases and the oxygen transport function is restored, the pain will be relieved naturally. Active antiviral therapy to restore the respiratory system is the key to alleviating the pain (68, 69). Under normal circumstances, patients with skeletal muscle joint pain can use non-steroidal anti-inflammatory drugs (NSAIDs) to relieve pain, and there is no evidence that NSAIDs aggravate COVID-19 (70). So, we may be able to relieve pain symptoms by administering NSAIDs. In addition to the important roles of vitamin D in bone metabolism, it is also involved in the progression of COVID-19. It was observed by Sulli A et al. that 25OH-vitamin D serum deficiency is associated with more severe pulmonary involvement, increased disease duration and mortality risk among elderly COVID-19 patients (71). It was also noted in a retrospective case-control study that compared with population-based controls, serums levels of 25OH-vitamin D are lower in hospitalized COVID-19 patients, who were at increased risk of Vitamin D deficiency (72). However, Vitamin D supplement among patients with COVID-19 is highly controversial. It was observed in a multi-center, randomized clinical trial that A 5000 IU daily oral vitamin D3 supplementation for 2 weeks decreased recovery times for cough and gustatory sensory loss among mild to moderate COVID-19 patients with poor vitamin D status (73). Meanwhile, it was also noted by Murai IH et al. in a randomized controlled trial that a single high dose of vitamin D3 did not significantly reduce hospitalization time for COVID-19 patients (74). Hence, so far, vitamin D supplement is recommended for everyone with increased time spent indoors due to strict lockdowns during COVID-19, only in order to maintain bone and muscle health, but there is not sufficient evidences to support vitamin D supplement with the aim of preventing or treating COVID-19 (75). Additionally, staying in bed in the ICU for a long time will cause an imbalance in protein synthesis and degradation, especially in the lower limbs, where muscle protein turnover is reduced, protein synthesis is slow, degradation increases, muscle cells atrophy, and strength decreases. Therefore, patients should minimize the time of intubation to reduce the time of bed rest and engage in appropriate activities to enhance the strength of the skeletal muscles. In SRAS-COV-1 from 2002 to 2004, studies have proved that rehabilitation exercise is beneficial to restore the function of the musculoskeletal system. Survivors of COVID-19, which has a similar pathogenesis, should be able to recover the function of their musculoskeletal system in the same way with the same rehabilitation exercises (76). In China, rehabilitation exercises have been added to treatment guidelines for COVID-19 patients to improve lung function via leisurely activities (77). In addition, pulmonary rehabilitation is also necessary for COVID-19 patients. It was demonstrated that different pulmonary rehabilitation interventions, including combined exercise (aerobic with strength), combined exercise with specific respiratory exercises, and aerobic exercises with specific respiratory muscle training, could obviously improve pulmonary functional and quality of life of patients recovered from severe COVID-19 (78). It was also recommended that rehabilitation care is crucial for patients with severe COVID-19, which should be implemented as soon as possible, and performed at the same time with regular treatment of COVID-19 according to the correct assessment (79).

## Effects of Anti-Inflammatory Drugs on Long-Term Sequelae of Covid-19

Dysregulated inflammatory response during SARS-CoV-2 infection was regarded to be major cause of severity and death of COVID-19 (80). Therefore, anti-inflammatory drugs are widely used during the treatment of COVID-19. In addition to its short-term benefits, use of anti-inflammatory drugs also affect long-term outcomes of COVID-19 patients. According to current studies, use of anti-inflammatory drugs, such as tocilizumab and NSAIDs, have no adverse effects on the short or long outcomes of COVID-19 (81, 82). In addition, it was demonstrated by Catalán IP et al. that treatment with systemic corticosteroids during hospital admissions is associated with decreased long-term symptoms and improved quality of life, which could be explained by relieving intense inflammation during the acute phase of COVID-19 and subsequently reduce organ and tissue damages caused by SARS-CoV-2 infection. (83). Due to extensive use of anti-inflammatory drugs in the treatment of COVID-19, risk of late-onset complications has always been concerned. Therefore, more long-term follow-up studies of COVID-19 patients treated with various anti-inflammatory drugs are necessary for safety assess and appropriate use of anti-inflammatory drugs among COVID-19 patients.

## Side Effects of the Covid-19 Vaccines

**Figure 1:**
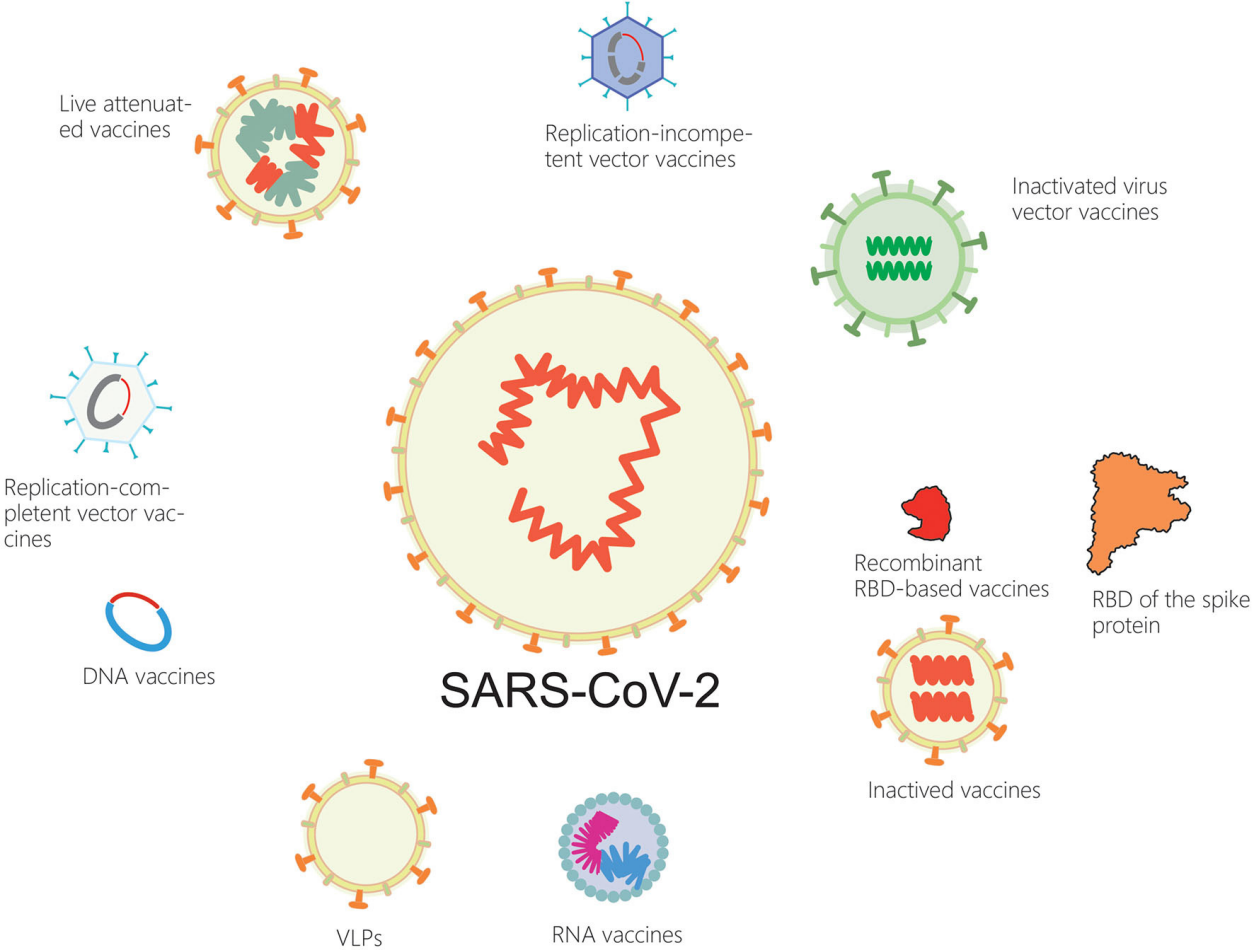
Current SARS-CoV-2 vaccines.

In the next few months, billions of people around the world will be vaccinated against the new coronavirus. Serval SARS-CoV-2 vaccine candidates are currently available, including inactivated virus vaccines, live attenuated vaccines, recombinant protein vaccines, DNA vaccines and RNA vaccines. Other types of vaccines include replication-incompetent vector vaccines, replication-competent vector vaccines, inactivated virus vector vaccines that display the spike protein on their surface (84) (Figure 1).

Ad5-nCoV, a viral vector vaccine which was developed by CanSino Biologics and also the People's Liberation Army, was the first candidate to participate in human trials and is now in clinical phase 2 (85, 86). Having proved to be safe in single blind, randomized phase 1/2 clinical trials, ChAdOx1 which was developed by scientists at Oxford University has already started phase 3 clinical trials (87). In Russia, a heterologous recombinant adenovirus (rAd)-based vaccine, Gam-COVID-Vac, manufactured by Gamaleya NRCEM (Moscow, Russia) started phase 3 trials and showed 91.6% efficacy against COVID-19 (88). In addition, a US-based biotechnology firm has developed a DNA-based vaccine named INO-4800, which is uses a relatively more recent vaccine technique. PiCoVacc, which is an inactivated vaccine developed by a Chinese private biopharma company, have currently entered phase 3 trial (89). BBIBP-CorV is also an inactivated vaccine in phase 1/2 clinical trials which is developed by Beijing Institute of Biological Products (Beijing, China) (90). The Beijing Institute of Biological Products and the Wuhan Institute of Biological Products are also developing some inactivated vaccines, which are in the 2/3 clinical phases. Recently, Clover Biopharmaceuticals (Chengdu, China) developed a s-trimer (S-Trimer) SCB-2019 with two different adjuvants, which triggered obvious humoral and cellular response to SARS-CoV-2 in clinical phase 1 (91). Moreover, two RNA vaccines named mRNA-1273 and BNT-162 are also under development, which could transmit information molecules to guide human body cells and produce the spike proteins of SARS-CoV-2 (92).

The World Health Organization (WHO) maintains a working document that includes most of the vaccines in development and it is available at https://www.who.int/publications/m/item/draft-landscape-of-covid-19-candidate-vaccines. The development of a mRNA vaccine to prevent SARS-CoV-2 infection was successful, and no serious problems occurred during the phase 3 clinical trials that are still ongoing (93). However, clinical trials of the vaccine have shown that the vaccine is not absolutely safe, and some recipients experience different side effects (94). The main symptoms include fever, arm soreness and fatigue. Most scientists think these symptoms are uncomfortable but not dangerous. Pfizer's COVID-19 vaccine shows 95% safety, but due to the large population receiving the vaccine, a 5% risk will cause hundreds of thousands of people to have different side effects. There are also occasional severe allergic reactions within a few hours after receiving Pfizer's COVID-19 vaccine. In addition, a vaccine from Australia was also urgently stopped due to false positives for HIV after vaccination. At present, vaccines designed in Europe and China have more advantages in safety, but lack extensive clinical trial data. Each vaccine has more or less unknown side effects and complication, and their long-term sequelae require clinical trials and the test of time. These severe allergic reaction cases raise more questions than the answers given and such safety signals are almost inevitable after a vaccination programme involving millions of people is launched. We need to develop a firm and proactive “safe road map” to clarify the pathogenesis, identify people at risk of such reactions, and adopt strategies that help with treatment and prevention.

## Discussion

The current study indicated that the complication and sequelae of COVID-19 are primarily caused by the patient's immune disorder. In addition, the clinical symptoms and prognosis of SARS-CoV-2 infection are also closely related to immune disorders. Most countries regard elevated IL-6 as a serious and critical clinical warning indicator. According to the clinical data of patients with COVID-19, pro-inflammatory cytokines and chemokines can avoid the risk of a cytokine storm and achieve the possibility of self-healing, which is also the reason why most mild and moderate patients can be cured (95). However, some mild patients also experienced a cytokine storm due to immune disorders. This involves a suicide attack that can damage the virus but also leave behind collateral damage, which will eventually lead to further lung injury, multiple organ failure and even increase the risk of death (96). Therefore, monitoring the level of pro-inflammatory cytokines and taking corresponding intervention measures as early as possible is of great significance to delay the transformation of mild or moderate patients to severe patients and also to achieve good clinical outcomes.

In addition to the therapeutic approaches mentioned above, inflammatory factor blockers (glycyrrhetinic acid, tocilizumab, interferon-γ blockers) and stem cell therapy are also expected to become effective means to control cytokines. Additionally, some Chinese medicine prescriptions can also block viruses from entering cells and regulate the body's immune response. Regarding the highly controversial method of reinfusing the serum of recovered COVID-19 patients, although most studies have confirmed that it can enhance immunity, it is difficult to obtain plasma during the recovery period, and its effectiveness may be related to the timing of its extraction. The neutralizing antibody titer in the serum is variable, so its clinical application is also subject to certain restrictions.

**Figure 2:**
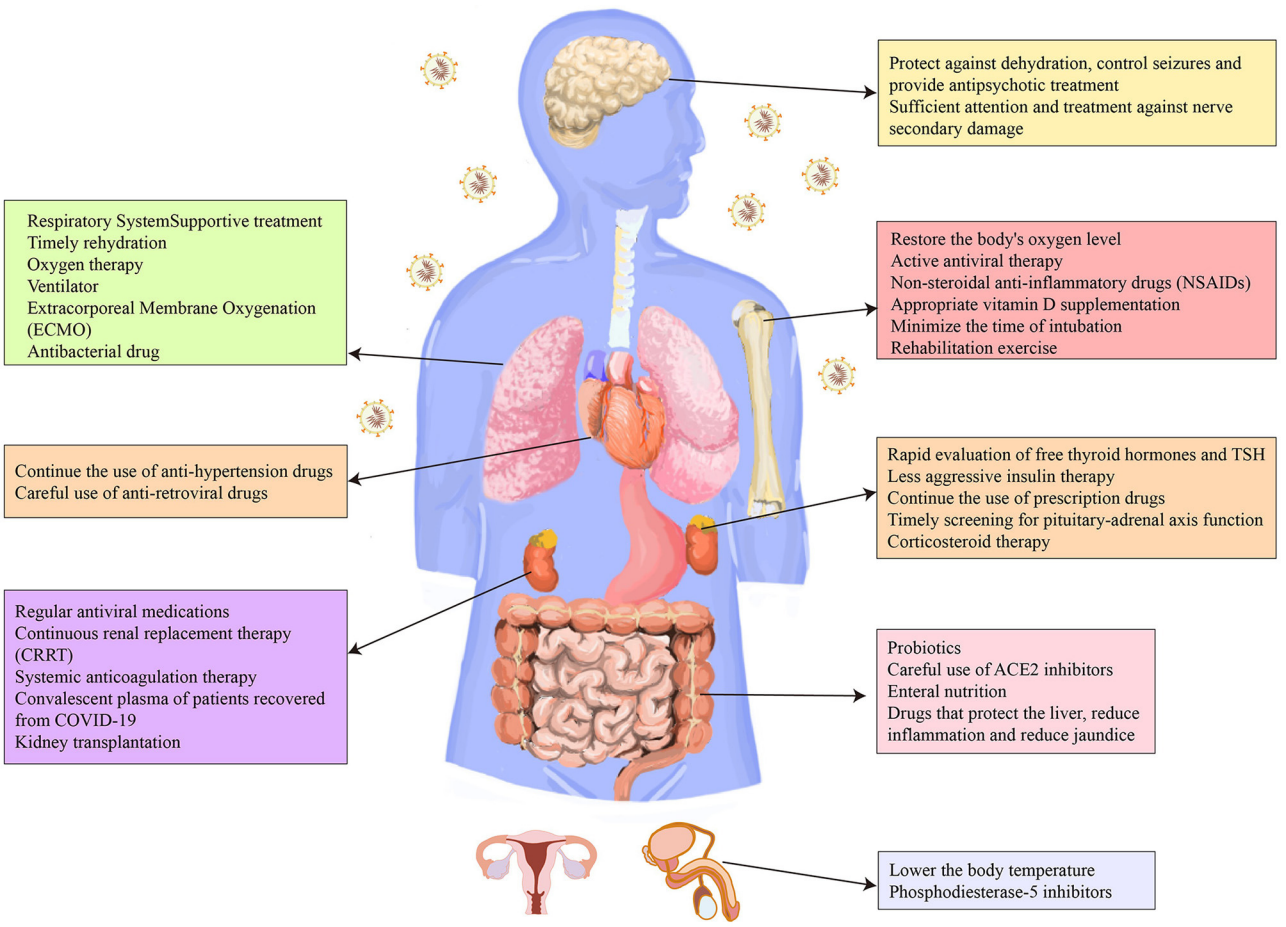
Therapeutic strategies for the complications and sequelae of eight major systems caused by COVID-19.

At present, the therapy of complications and sequelae of COVID-19 is limited to symptomatic treatment, and there is even no effective treatment for complications or sequelae in certain systems, such as the reproductive system. Therefore, more attention should be focused on the effectiveness and safety of the novel treatment measures of COVID-19, especially the treatment of the complications and sequelae. In addition, with the widespread vaccination of COVID-19 vaccines in various countries, medical workers should always pay attention to the side effects of the vaccine and give timely treatment. We systematically analyzed current treatment for the complications and sequelae of COVID-19 (Figure 2). We also sorted out the side effects reported in the vaccine trials. We believe that our research will provide a theoretical rationale for the management of COVID-19 survivors and thereby improve their prognosis.

## Author Contributions

Material preparation, data collection, and analysis were performed by JW, KZ, YX, GW, and LT. The first draft of the manuscript was written by JW, KZ, YX, and LT. All authors commented on previous versions of the manuscript, read and approved the final manuscript, and contributed to the study conception and design.

## Conflict of Interest

The authors declare that the research was conducted in the absence of any commercial or financial relationships that could be construed as a potential conflict of interest.

## Publisher's Note

All claims expressed in this article are solely those of the authors and do not necessarily represent those of their affiliated organizations, or those of the publisher, the editors and the reviewers. Any product that may be evaluated in this article, or claim that may be made by its manufacturer, is not guaranteed or endorsed by the publisher.
